# Computed tomography findings in 11,504 adult patients with traumatic brain injury: a large real-world cohort study with a S100B subgroup analysis

**DOI:** 10.1007/s00402-026-06397-y

**Published:** 2026-06-30

**Authors:** Clemens Clar, Paul Puchwein, Maximilian Moshammer, Patrick Sadoghi, Diether Kramer, Andreas Leithner, Patrick Reinbacher

**Affiliations:** 1 Department of Orthopedics and Traumatology, Medical University of Graz, Graz, Austria; 2 Division of Plastic, Aesthetic and Reconstructive Surgery, Medical University of Graz, Graz, Austria

**Keywords:** Traumatic brain injury, S100B level, Biomarkers

## Abstract

**Objective:**

The aim of this study was to characterize acute cranial CT findings in a large cohort of adult patients with traumatic brain injury and to explore the diagnostic performance of S100B in a selected subgroup. The hypothesis was that most cranial CT scans in adult patients with traumatic brain injury would not demonstrate acute traumatic abnormalities, and that, within a selected subgroup, negative S100B levels would be associated with the absence of such findings on CT.

**Methods:**

This retrospective cohort study included 11,504 adult patients presenting with traumatic brain injury to a level I trauma center between 04/2016 and 05/2024. All patients underwent cranial CT, while serum S100B measurements were available in a selected subset. CT findings were classified as normal or showing acute traumatic abnormalities. In the S100B subgroup, biomarker levels were analyzed in relation to CT findings to assess their diagnostic performance for excluding acute pathology.

**Results:**

A total of 11,504 adult patients with traumatic brain injury were included; 27.4% were inpatients and 72.6% outpatients. Overall, 81.9% of CT scans showed no acute pathology, while 10.7% demonstrated abnormalities. 7.4% of all cases were excluded. Acute TBI-related interventions were rare (3.7% of inpatients vs. 0.3% of outpatients). In patients with dementia and those receiving anticoagulation, CT scans remained normal in 81.7% and 81.9%, respectively. S100B was available in 483 patients (4.2%); negative values were associated with absence of CT pathology, with higher negative predictive value in outpatients (98%) than in inpatients (73%) (*p* < 0.001).

**Conclusion:**

In this large retrospective cohort of adult patients with traumatic brain injury, most cranial CT examinations were negative for acute pathology. In a selected subgroup, negative S100B levels were associated with the absence of CT abnormalities, with substantially lower performance in inpatients. These findings suggest a potential adjunctive role of S100B in selected low-risk patients, however further prospective studies with standardized testing protocols are required to define the diagnostic utility and clinical applicability of S100B more precisely in the evaluation of traumatic brain injury.

## Introduction

Traumatic brain injury (TBI) represents a major global health burden, with more than 10 million cases reported annually worldwide. Epidemiological data from European populations demonstrate a marked age-dependent increase in incidence, with substantially higher rates observed in elderly individuals, who are also characterized by a higher prevalence of comorbidities such as dementia and cardiovascular disease. This demographic shift has important clinical implications, as older patients are not only at increased risk of sustaining TBI, but also present unique diagnostic challenges due to altered clinical presentation and limited reliability of neurological assessment. Cranial computed tomography (CT) remains the cornerstone of diagnostic evaluation in patients with suspected TBI, given its high sensitivity for detecting acute intracranial pathology. Consequently, CT imaging is widely used in emergency departments, often with a low threshold for indication. While this strategy prioritizes diagnostic safety, it also results in a substantial proportion of examinations without clinically relevant findings, contributing to increased resource utilization, prolonged emergency department workflows, and cumulative radiation exposure [1–3]. 

To optimize imaging utilization, several clinical decision rules, including the Canadian CT Head Rule and the New Orleans Criteria, have been developed and validated, demonstrating high sensitivity for identifying patients at risk of intracranial injury. However, their application may be limited in specific patient populations, particularly in older adults and those with cognitive impairment, in whom clinical assessment is frequently less reliable and thresholds for imaging are consequently lower [1, 3, 4]. 

In this context, serum biomarkers have emerged as potential adjunctive tools to support clinical decision-making. Among these, S100B, a calcium-binding protein predominantly expressed in astroglial and Schwann cells, is released into the circulation following brain injury and has been investigated as a marker of intracranial pathology [5–8]. Previous studies have suggested that S100B may be useful in ruling out clinically relevant brain injury in selected patients, and its use has been incorporated into certain guideline recommendations, such as those of the Scandinavian Neurotrauma Committee. These approaches indicate that biomarker-guided triage may reduce the number of unnecessary CT scans in carefully selected populations [9–13]. 

To date, large-scale real-world data integrating cranial CT findings with biomarker-based assessment in adult traumatic brain injury remain limited. In this context, a systematic evaluation between serum S100B levels and acute CT findings in a large consecutive cohort, while simultaneously characterizing imaging utilization patterns in routine clinical practice was conducted.

The aim of this study was to characterize acute cranial CT findings in a large cohort of adult patients with traumatic brain injury and to explore the diagnostic performance of S100B in a selected subgroup. The hypothesis was most cranial CT scans in adult patients with traumatic brain injury would not demonstrate acute traumatic abnormalities, and that, within a selected subgroup, negative S100B levels would be associated with the absence of such findings on CT.

## Materials and methods

### Study design and participants

This retrospective single-center cohort study evaluated adult patients presenting with TBI to the emergency department of a level I trauma center between April 2016 and May 2024. All relevant data were retrieved from the institutional hospital information system. The study aimed to characterize acute cranial CT findings in a large real-world TBI cohort and to examine the diagnostic rule-out performance of serum S100B in the subgroup of patients with an available valid biomarker measurement.

### Inclusion and exclusion criteria

Patients were eligible if they were at least 18 years of age and presented during the study period with documented TBI and a cranial CT examination with or without a valid S100B measurement. Patients were excluded if presentation occurred outside the study period, if CT or S100B documentation was insufficient, or if the minimum age criterion was not met. CT examinations with motion artefacts precluding reliable interpretation or erroneous coding were also excluded from the respective analyses.

### Data acquisition and variable definition

Patient-level data were extracted from the hospital information system and reviewed by two independent assessors to reduce extraction errors. Structured variables included age, sex, documented diagnoses, treatment status, anticoagulation status, dementia, acute TBI-related interventions, CT findings, and S100B measurements when available. Documented TBI was defined by the presence of an ICD-coded diagnosis consistent with traumatic brain injury or head trauma in the institutional hospital information system. The ICD-10 codes used for case identification included S00.- superficial injury of head, S01— open wound of head, S02— fracture of skull and facial bones, S06— intracranial injury, S07— crushing injury of head, S08— traumatic amputation of part of head, and S09— other and unspecified injuries of head. The ICD-based case identification was used to identify potentially eligible patients and was subsequently cross-checked against the available clinical and radiological documentation.

Treatment pathway was defined according to the documented disposition following the index emergency department presentation. Patients were classified as outpatients if they were evaluated and managed in the emergency department or ambulatory trauma setting and discharged without formal hospital admission after completion of the initial diagnostic workup. Patients were classified as inpatients if they were admitted to a hospital ward, intermediate care unit, or intensive care unit for further observation, treatment, or intervention related to the index traumatic brain injury episode. A patient was assigned to the anticoagulation subgroup if anticoagulant therapy had been recorded in the hospital information system or could be confirmed from the medical history. Included agents comprised coumarins, heparins and related substances, synthetic pentasaccharide factor Xa inhibitors, direct oral anticoagulants, direct factor Xa inhibitors, and direct thrombin inhibitors. The dementia subgroup included patients with a previously documented diagnosis of dementia or a diagnosis established by a specialist. Acute TBI-related procedures, including craniotomy, intracranial pressure probe placement, intubation, and other interventions, were identified from the hospital information system.

### Clinical workflow and S100B assessment

In routine clinical practice, patients without acute life-threatening conditions underwent physician assessment followed by blood sampling for S100B measurement. A positive S100B result was followed by urgent CT imaging. Patients with negative S100B values also underwent further clinical assessment and CT according to the treating physician’s judgment. Patients receiving anticoagulation underwent immediate CT because of the increased risk of intracranial hemorrhage. Patients with unstable vital signs or altered neurological status underwent urgent CT first, with S100B measurement obtained thereafter when applicable. Radiological findings were reported by experienced radiologists and repeatedly cross-checked.

For S100B analysis, blood was drawn into EDTA tubes. Samples were eligible only when obtained between 30 min and 6 h after trauma. Samples were centrifuged for 10 min, analyzed within 20 min in the institutional laboratory, and electronically transferred to the hospital information system. The threshold for a positive S100B result was predefined as ≥ 0.105 µg/L. For the diagnostic performance analysis of S100B, cranial CT served as the radiological reference standard for the presence or absence of acute traumatic cranial pathology. Therefore, only patients with both a valid S100B measurement and an available cranial CT classification were included in this analysis.

### CT classification

CT findings were categorized using a predefined three-level classification:

0 = no acute traumatic cranial pathology;

1 = acute intracranial hemorrhage (ICH) with or without recent skull fracture.

2 = recent skull fracture without ICH.

For analyses of diagnostic performance, CT categories 1 and 2 were considered acute traumatic CT abnormalities, whereas category 0 was considered a negative CT result.

### Outcome

The primary outcome was the prevalence of acute traumatic cranial CT abnormalities in the overall TBI cohort, defined as either acute ICH with or without skull fracture or isolated recent skull fracture. The co-primary outcome in the biomarker subgroup was the diagnostic rule-out performance of S100B for acute traumatic CT abnormalities, assessed by the proportion of negative S100B results among patients without acute CT pathology and summarized as negative predictive value (NPV). Sensitivity of S100B for CT-detected traumatic abnormalities was analyzed as an additional diagnostic performance parameter.

Secondary outcomes were distribution of CT findings according to inpatient versus outpatient treatment status; distribution of CT findings in patients with documented anticoagulation; distribution of CT findings in patients with documented dementia; age and sex distributions across treatment groups and the frequency of acute TBI-related interventions.

### Statistical analysis

The dataset was compiled into a spreadsheet using Excel^®^ (Microsoft^®^ Corporation, Redmond, WA, USA) for statistical evaluation. Data analysis was subsequently performed with IBM^®^ SPSS^®^ Statistics, version 29.0 (IBM Corp., Armonk, NY, USA). Descriptive statistics were used to summarize demographic, clinical, radiological, and biomarker-related variables. Group comparisons were performed using Pearson’s chi-square test or Fisher’s exact test, as appropriate. In the S100B subgroup, diagnostic performance was assessed using cranial CT as the reference standard, and sensitivity and negative predictive value were calculated for acute traumatic CT abnormalities. Statistical significance was set at *p* < 0.05.

## Results

### Study population

A total of 11,504 adult TBI cases were included. Of these, 3,157 patients (27.4%) were managed as inpatients and 8,347 (72.6%) as outpatients. Across the overall cohort, 9,419 CT examinations (81.9%) showed no acute traumatic cranial pathology, whereas 1,230 examinations (10.7%) demonstrated acute abnormalities. Specifically, 664 patients (5.8%) had ICH with or without a recent skull fracture, and 566 patients (4.9%) had a recent skull fracture without ICH. A total of 855 CT examinations (7.4%) were excluded because they did not meet the predefined criteria for analysis (Table 1).

**Table 1:** Overview of CT findings, demographic characteristics, and patient distribution in the inpatient and outpatient cohorts, including rates of pathological findings, exclusions, gender distribution, and age parameters across the entire study population

Parameter	Inpatients (n = 3,157)	Outpatients (n = 8,347)	Total (n = 11,504)
Normal CT	1,833 (58.1%)	7,586 (90.9%)	9,419 (81.9%)
ICH ± Fracture	605 (19.2%)	59 (0.7%)	664 (5.8%)
Fracture without ICH	301 (9.5%)	265 (3.2%)	566 (4.9%)
Excluded	418 (13.2%)	437 (5.2%)	855 (7.4%)
Pathological CTs total	906 (28.7%)	324 (3.9%)	1,230 (10.7%)
Male	1,850 (58.6%)	3,814 (45.7%)	5,664 (49,2%)
Female	1,307 (41.4%)	4,533 (54.3%)	5840 (50,8%)
Median age	64 years	74 years	74 years
Mean age	60.7 years	65.6 years	64.3 years

### CT findings according to treatment status

**Fig. 1:**
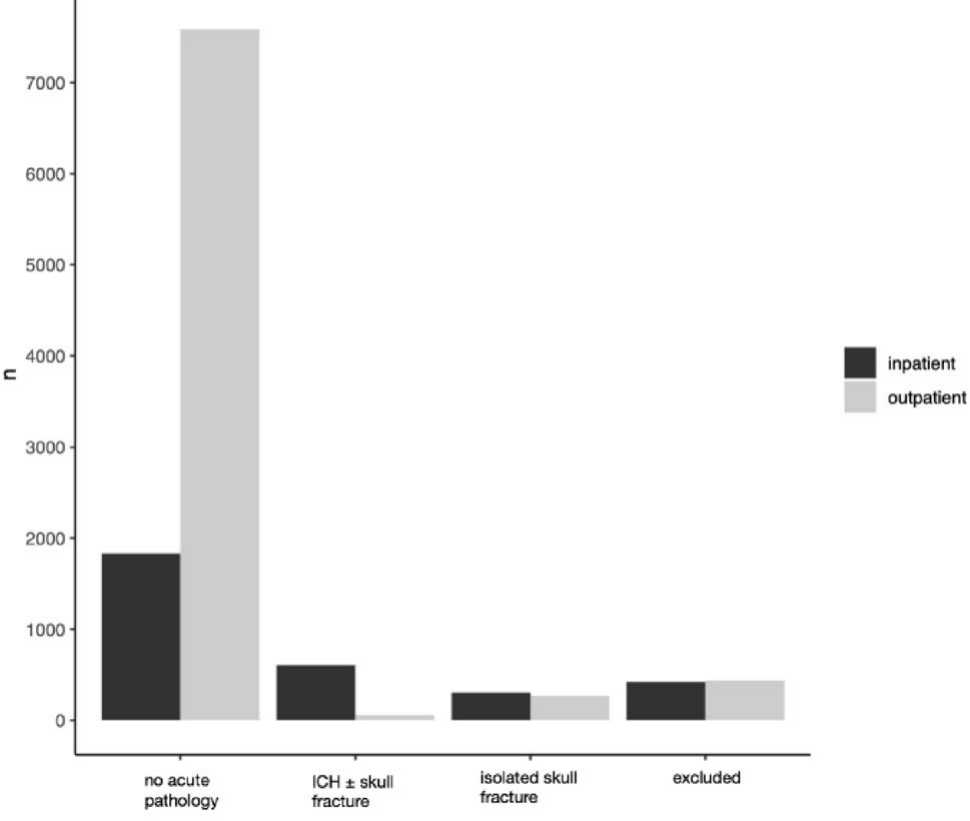
Absolute frequency of the 11,504 CT findings categorized in outpatient and inpatient cohorts

Marked differences in CT findings were observed between inpatients and outpatients. Among inpatients, 1,833 CT examinations (58.1%) were normal, 605 (19.2%) showed ICH with or without recent skull fracture, and 301 (9.5%) showed recent skull fracture without ICH. In addition, 418 examinations (13.2%) were excluded. In contrast, among outpatients, 7,586 CT examinations (90.9%) were normal, 59 (0.7%) showed ICH with or without skull fracture, and 265 (3.2%) showed recent skull fracture without ICH. 437 examinations (5.2%) were excluded (Fig. 1).

### Demographic characteristics

Sex distribution differed between treatment groups. Among inpatients, 1,850 patients (58.6%) were male and 1,307 (41.4%) were female. Among outpatients, 3,814 patients (45.7%) were male and 4,533 (54.3%) were female. Regarding age, inpatients had a median age of 64 years and a mean age of 60.7 years, whereas outpatients had a median age of 74 years and a mean age of 65.6 years.

### Acute TBI-related procedures

Documented acute TBI-related procedures were uncommon overall but were concentrated in the inpatient setting. Among outpatients, 28 of 8,347 patients (0.3%) had recorded acute TBI-related procedures. Among inpatients, 116 of 3,157 patients (3.7%) underwent documented acute TBI-related procedures.

### Dementia subgroup

Among 1,445 patients with pre-existing dementia, 1,180 CT examinations (81.7%) were normal. Acute traumatic abnormalities were identified in 111 patients (7.7%), including 63 cases (4.4%) with ICH with or without skull fracture and 48 cases (3.3%) with skull fracture without ICH. A further 154 examinations (10.7%) were excluded.

### Anticoagulation subgroup

Among 3,920 patients receiving known anticoagulation, 3,211 CT examinations (81.9%) were normal. Acute traumatic abnormalities were present in 353 cases (9%), comprising 200 cases (5.1%) with ICH with or without skull fracture and 153 cases (3.9%) with isolated skull fracture without ICH. A total of 356 examinations (9.1%) were excluded.

### S100B subgroup

Of the 11,504 total cases, 483 (4,2%) patients had a valid S100B measurement available for analysis. This subgroup comprised 232 men (48%) and 251 women (52%). Overall, 172 patients (35.6%) were treated as inpatients and 311 (64.4%) as outpatients. In the inpatient S100B subgroup, the median and mean age were both 52 years. In the outpatient S100B subgroup, the median age was 32 years and the mean age was 39 years (Table 2).

**Table 2:** Baseline demographic and clinical characteristics according to availability of valid S100B measurement

Variable	Overall cohort (n = 11,504)	Valid S100B measurement (n = 483)	No valid S100B measurement (n = 11,021)
Mean age	64.3	43.6	65.2
Male sex, n (%)	5,664 (49.2%)	232 (48.0%)	5,432 (49.3%)
Female sex, n (%)	5,840 (50.8%)	251 (52.0%)	5,589 (50.7%)
Inpatient treatment, n (%)	3,157 (27.4%)	172 (35.6%)	2,985 (27.1%)
Outpatient treatment, n (%)	8,347 (72.6%)	311 (64.4%)	8,036 (72.9%)

**Fig. 2:**
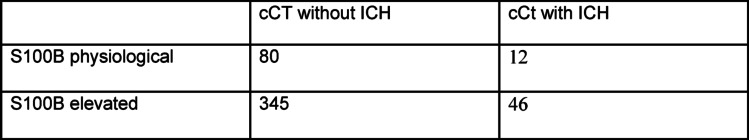
Confusion matrix comparing S100B levels with CT findings in TBI patients of the entire cohort

Within this subgroup, S100B showed different diagnostic rule-out performance according to treatment status. In outpatients, the negative predictive value of S100B for excluding acute traumatic CT abnormalities was 98%, with a sensitivity of 75%. In inpatients, the corresponding NPV was 73%, with a sensitivity of 80%. Both associations were statistically significant (*p* < 0.001). Mean S100B concentration was 0.067 µg/L in patients with negative S100B values and 0.573 µg/L in those with positive results (Fig. 2).

##  Discussion

The most important findings of this study are that most cranial CT examinations in adult patients with traumatic brain injury did not reveal acute traumatic abnormalities and that, within a selected subgroup, negative S100B levels were associated with the absence of CT-detected pathology. Notably, the diagnostic performance of S100B differed substantially between clinical settings, with markedly higher rule-out capability observed in outpatients compared to inpatients.

The high proportion of normal CT findings observed in this large real-world cohort underscores the well-recognized discrepancy between clinical suspicion and radiologically confirmed traumatic pathology in adult TBI. In the present analysis, more than four out of five CT examinations were negative, a finding that remained consistent across clinically vulnerable subgroups, including patients with dementia and those receiving anticoagulation. While these observations do not imply inappropriate imaging, they highlight the inherent challenges in clinical triage of TBI, where a low threshold for imaging is often maintained to avoid missing clinically significant intracranial injury. In this context, interest in adjunctive diagnostic strategies, including serum biomarkers such as S100B, has increased. In the present study, S100B demonstrated a high negative predictive value in the outpatient subgroup, aligning with current literature, suggesting potential utility as a rule-out tool in selected lower-risk patients. In this context, lower-risk patients primarily refer to patients with mild traumatic brain injury or minor head injury, commonly characterized by a Glasgow Coma Scale score of 13–15, absence of focal neurological deficits, and no major high-risk features on initial clinical assessment. Previous studies and guideline-based evaluations have shown that incorporation of S100B into the diagnostic workup of such mild TBI populations may reduce cranial CT utilization by approximately one-third without compromising the detection of clinically relevant intracranial injury [7, 8]. However, this performance was not replicated in inpatients, in whom the negative predictive value was substantially lower. This divergence is clinically relevant and likely reflects differences in baseline risk, patient selection, and pretest probability of intracranial injury. Patients requiring hospitalization typically present with more severe symptoms, higher comorbidity burden, or more concerning injury mechanisms, all of which reduce the reliability of a biomarker-based rule-out approach [15–17]. 

From a clinical perspective, this finding is particularly relevant in the context of cumulative radiation exposure. Although the effective dose of a single cranial CT is relatively low, typically ranging between 1 and 3 mSv, repeated imaging and large-scale utilization contribute to a measurable increase in lifetime malignancy risk [16]. This risk is dose-dependent and correlates with both the number of performed scans and cumulative exposure over time [18, 19]. Given that the majority of CT examinations in the present cohort were negative, a substantial proportion of patients were exposed to ionizing radiation without evidence of acute traumatic pathology. These findings support ongoing efforts to refine clinical decision-making and to further develop and implement validated imaging guidelines and risk-adapted algorithms aimed at reducing unnecessary CT utilization [20, 21]. Beyond radiation concerns, the widespread use of CT imaging also carries important economic implications and contributes to resource constraints, particularly in high-volume emergency settings with limited radiological capacity.

The subgroup analyses provide additional insight into specific patient populations that may contribute disproportionately to imaging utilization. In patients with pre-existing dementia, the proportion of CT scans demonstrating acute pathology was lower than in the overall cohort. This finding likely reflects the inherent diagnostic challenges in this population, where baseline cognitive impairment may obscure clinical assessment and lead to a lower threshold for imaging. Overlapping symptoms between dementia and TBI, combined with communication barriers, may result in a more cautious diagnostic approach, including liberal CT use. While this strategy prioritizes patient safety, it may also contribute to increased imaging rates despite a low prevalence of radiologically confirmed injury. These findings suggest that tailored diagnostic pathways or modified clinical decision rules may be beneficial in this subgroup, although any attempt to reduce imaging must be balanced against the risk of missed intracranial pathology [22]. 

A similar pattern was observed in patients receiving anticoagulation therapy. Despite the known increased risk of intracranial hemorrhage in this population, the overall proportion of acute CT abnormalities remained relatively low. This apparent discrepancy likely reflects a risk-averse diagnostic strategy, with a low threshold for imaging driven by the potentially severe consequences of missed bleeding events. Current clinical decision rules, including the Canadian CT Head Rule and the New Orleans Criteria, do not uniformly account for all aspects of anticoagulation status, and their applicability in this subgroup remains limited. The present findings therefore support the need for refined or adapted decision algorithms that incorporate anticoagulation as a key risk modifier while maintaining diagnostic safety [4, 23]. 

The comparison between inpatient and outpatient populations further emphasizes the role of clinical context in imaging decisions. Inpatients were younger and exhibited a substantially higher proportion of acute CT abnormalities, consistent with a higher baseline risk profile. However, it is noteworthy that more than half of hospitalized patients still had no detectable traumatic pathology on CT. This highlights the complexity of clinical triage in TBI and suggests that current strategies may lack sufficient specificity. At the same time, this observation should not be interpreted as evidence of inappropriate imaging, but rather as a reflection of the prioritization of sensitivity over specificity in emergency diagnostics.

Taken together, these findings reinforce the need for improved, context-sensitive diagnostic approaches in TBI. While established clinical decision rules provide a valuable framework, their performance may be limited in specific subgroups such as elderly patients, those with cognitive impairment, or patients receiving anticoagulation. Future research should therefore focus on the development and validation of integrated diagnostic pathways that combine clinical assessment, risk stratification tools, and potentially adjunctive biomarkers, with the aim of optimizing both diagnostic safety and resource utilization.

### Limitations

This study has several limitations. First, its retrospective single-center design limits external validity and is subject to documentation bias. Second, S100B measurements were available in only 4.2% of patients, introducing substantial selection bias and limiting generalizability. Third, biomarker testing was not applied systematically, precluding robust assessment of diagnostic accuracy. Fourth, incomplete availability of full contingency data restricted reporting of specificity and positive predictive value. Fifth, detailed timing of S100B sampling relative to injury was not consistently available. Finally, CT-based classification does not capture all forms of traumatic brain injury, particularly lesions not detectable on routine CT.

## Conclusion

In this large retrospective cohort of adult patients with traumatic brain injury, most cranial CT examinations were negative for acute pathology. In a selected subgroup, negative S100B levels were associated with the absence of CT abnormalities, with substantially lower performance in inpatients. These findings suggest a potential adjunctive role of S100B in selected low-risk patients, however further prospective studies with standardized testing protocols are required to define the diagnostic utility and clinical applicability of S100B more precisely in the evaluation of traumatic brain injury.

## Data Availability

The data that support the findings of this study are not openly available due to reasons of sensitivity but are available from the corresponding author upon reasonable request.

## Abbreviations

CT: Computed tomography
ICH: Intracranial hemorrhage
L: Litre
NPV: Negative predictive value
TBI: Traumatic brain injury
µg: Microgram
